# Evaluation of potential role of vitamin C in differential skeletal development between female and male mice using a mouse model

**DOI:** 10.1186/1471-2105-13-S12-A18

**Published:** 2012-07-31

**Authors:** Yan Jiao, Yonghui Ma, Xiaoyun Liu, Weikuan Gu

**Affiliations:** 1 Department of Orthopedic Surgery – Campbell Clinic, University of Tennessee Health Science Center, Memphis, TN 38163, USA

## Background

In humans, gender difference has been suggested by previous studies. Sex difference in vitamin C requirements have also been reported from animal models. Mouse spontaneous fracture model (sfx) lacks L-gulonolactone oxidase (LGO), an essential gene for the synthesis of ascorbic acid (Vitamin C). We have been using sfx model for the past eight years and we are the first group to identify the LGO mutation in this mouse model.

## Materials and methods

In order to identify the genes that regulate skeletal development through Vitamin C (VC) pathway, we analyzed the gene expression profile in mouse femur. For microarray analysis, femur from three age-matched, wild-type +/+ Balb/By, inbred strain mice (WT) and 3 female and 3 male homozygous sfx/sfx mice were used.

## Results

Myogenic factor 6 (Myf6) gene is differentially regulated by VC in male versus female femurs while Myf5 and Pax6 are almost undetectable in the mouse femur. Expression of Myf6 in female mice measured by DNA microarray experiment Myf6 is much down regulated. Myf6 is up regulated in femurs of male sfx mice. Myf6 is specifically expressed in skeletal system (femur) while in liver and kidney Myf6 is not expressed. There are sex differences in expression levels of collagen synthesis genes. There are sex differences between expression levels of hormone relevant genes. The differential expression of Myf6 was confirmed by real time PCR.

## Conclusions

Vitamin C may differentially regulate myf6 and collagen expression in the skeletal development through sex hormones and relevant factors.

